# Exogenous Nucleotides Ameliorate Ageing-Related Intestinal Inflammation in Senescence-Accelerated Mouse Prone-8 (SAMP8) Mice

**DOI:** 10.3390/nu15112533

**Published:** 2023-05-29

**Authors:** Mei You, Rui Liu, Chan Wei, Xiujuan Wang, Xiaochen Yu, Zhen Li, Ruixue Mao, Jiani Hu, Na Zhu, Xinran Liu, Rui Fan, Yong Li, Meihong Xu

**Affiliations:** 1 Department of Nutrition and Food Hygiene, School of Public Health, Peking University, Beijing 100191, China; 1610306135@pku.edu.cn (M.Y.);; 2 Beijing Key Laboratory of Toxicological Research and Risk Assessment for Food Safety, Peking University, Beijing 100191, China

**Keywords:** exogenous nucleotides, intestine, inflammation, immunity

## Abstract

As one of the most important barriers in the body, the intestinal barrier is a key factor in maintaining human health. Ageing of the intestine is a degenerative process that is closely associated with a variety of poor health conditions in the elderly. Inflammation and the immune system are anti-ageing targets that can regulate the function of the intestine. Nucleotides (NTs) are involved in important physiological and biochemical reactions in the body, but there are few studies about their effect on the ageing intestine. This paper examines the role of exogenous NTs in the ageing intestine. For this purpose, we used senescence-accelerated mouse prone-8 (SAMP8) mice and senescence-accelerated mouse resistant 1 (SAMR1) mice for the experiment, and randomly divided the mice into NTs-free, Normal Control, NTs-low, NTs-medium, NTs-high, and SAMR1 groups. After 9 months of intervention, we collected the colon tissue of mice for testing. In our study, exogenous NTs could increase bodyweight of mice during ageing and improve the morphological structure of the intestine, and we found that NTs could promote the secretion of intestinal protective factors, such as TFF3 and TE. Furthermore, supplementation with NTs suppressed intestinal inflammation and improved intestinal immunity, possibly by activating the p38 signaling pathway. These results suggest that exogenous NTs are able to maintain the health condition of the ageing intestine.

## 1. Introduction

The problem of an ageing population has become a global issue, which now makes anti-ageing an urgent research field. Ageing of the intestine is the phenomenon of structural degeneration and functional decline of the intestine over time. In the ageing intestine, certain pathological changes occur [1]: the secretion of the intestine decreases [2], intestinal immunity declines, etc. Deficiency of intestinal immunity makes it less resistant to pathogens, and it may cause the development of diarrhea and a high incidence of intestinal infections [3]. Previous studies have agreed that significantly increased intestinal permeability with ageing makes it easier for antigens and dietary origin to leak from the intestinal lumen into the circulation, which elevates the secretion of inflammatory factors and leads to low-level intestinal inflammation [4]. The decreasing level of digestion, absorption, and motility of the ageing intestine may give rise to intestinal diseases and malnutrition [5,6]. The functional ageing of the intestine contributes to the development of some degenerative and chronic illnesses [7,8], and it also increases the burden of diseases. Therefore, slowing down the ageing process of the intestine is an important aspect of maintaining normal health conditions in the elderly.

Chronic low-grade inflammation is thought to be a key marker of ageing. Expression of inflammatory genes is enhanced and pro-inflammatory proteins are upregulated during ageing [9]. Some studies have shown that pro-inflammatory cytokines increase in the ageing intestine [2], and that they ultimately disrupt the intestinal barrier. Furthermore, the destruction of the intestinal barrier may generate inflammatory diseases and have adverse consequences far beyond the intestine, leading to weakness and systemic disease [10]. There are many theories about intestinal ageing. The accumulation of DNA damage in the cellular genome promotes the ageing process and activates inflammatory signaling pathways [11]. The intestinal flora changes due to ageing, and it becomes an important factor in accelerating inflammation in vivo [4]. The innate immune system is activated by inflammation in most cases [12], and the body fights and clears pathogens in vivo and in vitro by activating the immune system [13]. Inflammation and the immune system interreact mutually, which is vital for maintaining homeostasis in the body.

Nucleic acids are bioinformatic macromolecules that can be found in deoxyribonucleic acid (DNA) and ribonucleic acid (RNA). Nucleotides (NTs) are the basic components of nucleic acids, and they can be involved in the transmission of genetic information. NTs are composed of bases, pentose, and phosphate. Dissociative NTs or their derivatives participate in the regulation of protein function and internal metabolism. In addition, dissociative NTs are major high-energy compounds in energy metabolism and are important messengers in cell signaling transduction. The ability to synthesize NTs from scratch reduced when the organism is under special circumstances, such as rapid growth, stress, and ageing [14]. Therefore, obtaining NTs externally is a better mode of supplementation. Exogenous NTs are beneficial for health—for instance, they improve immunity [15] and liver function [16], promote infant growth and development [17], etc. In contrast, deficiency of exogenous NTs may cause adverse effects. Exogenous NTs are important for intestinal health, too. The deficiency of dietary NTs leads to reduced protein synthesis in the intestine [18]. On the contrary, an exogenous NTs supplement can promote intestinal repair [19] and regulate the intestinal flora [20,21]. Intestinal mucosa lacks the ability to produce NTs [22], so supplementation with NTs is important for intestinal health. Exogenous NTs can reduce the level of intracellular inflammatory phenotypes [23], and can also suppress inflammation in vivo [24,25].

In this study, we aimed to explore how exogenous NTs influence the ageing intestine by using senescence-accelerated mouse prone-8 (SAMP8) and senescence-accelerated mouse resistant 1 (SAMR1) mice. Senescence-accelerated mice (SAM) include senescence-accelerated prone (SAMP line) mice and senescence-accelerated resistant (SAMR line) mice [26]. SAMP8 belongs to the SAM strain, and is widely used in ageing-related studies. The 6-month-old SAMP8 mice begin to show signs of intestinal motility dysfunction [27], and the 7-month-old SAMP8 mice have a significant decrease in intestinal function [28]. The intestine of the aged SAMP8 mice exhibit intestinal barrier damage, intestinal inflammation, and intestinal immune damage, etc. [9,29]. SAMP8 mice have already been used as an animal model in order to study ageing intestinal function [28,29], and SAMR1 mice were used as the model control here because of their normal ageing characteristics. In summary, we performed a 9-month dietary intervention with different doses of exogenous NTs to investigate their effect on the ageing intestines of mice, and we mainly focused on their effect on inflammation and immunity. Meanwhile, we performed a 9-month intervention without exogenous NTs in order to explore the role of NTs in the diet.

## 2. Materials and Methods

### 2.1. Test Substances

Exogenous NTs mixture (5′AMP:5′CMP:5′GMPNa_2_:5′UMPNa_2_ = 16:41:19:24) is produced from sucrose molasses by enzymatic degradation with a purity of >99%. Exogenous NTs used in this experiment were from Zhen-Ao Biotechnology Co., Ltd. (Dalian, China).

### 2.2. Animals and Treatment

Male 10–12-week-old SPF SAMP8 and SAMR1 mice were purchased from the Department of Experimental Animal Science, Peking University. Every single cage was provided for the mice with an environment of 12 h alternating lighting. The temperature was 24 ± 2 °C, and relative humidity was 50–60%. The mice were allowed to feed and drink freely during rearing. The mice and fodder were weighed weekly.

After 1 week of feeding for adaption, all SAMP8 mice were grouped into 15 mice as follows: NTs-free (NTs-F), normal control (Control), NTs-low (NTs-L), NTs-medium (NTs-M), and NTs-high (NTs-H) groups. Meanwhile, 15 SAMR1 mice were set as the SAMR1 model control group (SAMR1). Standard food (American Institute of Nutrition Rodent Diets-93M) was prepared for the control and SAMR1 groups, while standard food without NTs was prepared for the NTs-F group. Exogenous NTs were added to the standard food at different doses for the three NTs intervention groups. Descriptions of the groups and the fodder and the animals’ number are shown in Table 1. After a 9-month intervention, the colon tissue of the mice was collected and put in a −80 °C refrigerator.

### 2.3. Histomorphology Observation of the Intestine

Hematoxylin-eosin (H&E) was used in intestinal dewaxed sections for staining, and the morphological structure of colon tissue was observed and photographed under a microscope (BX43F, Olympus, Tokyo, Japan). The number of lymphocytes and goblet cells were recorded by randomly counting 100 cells from the intestinal villi in each section.

### 2.4. ELISA Analysis

We used ELISA analysis to test the levels of trefoil factor 3 (TFF3), telomerase (TE), IgA, IL-2, TNF-α, MCP-1, and CXCL-1 by using a 10% colon tissue homogenate [1,29]. We made a fitted curve for the standards and calculated the concentration of the sample. The TFF3 assay kit was obtained from Wuhan BOSTER, the TE activity assay kit was obtained from Jiang Sofia Biotechnology Co., Ltd. (Taizhou, China). The IgA, IL-2, MCP-1, and CXCL-1 assay kit were obtained from Hangzhou Multisciences, and the TNF-α assay kit from Invitrogen, Waltham, MA, USA. All of these indicators were measured according to the protocols provided with the assay kits.

### 2.5. Western Blot Analysis

The total protein of mice colon tissue was extracted and the supernatant of the homogenate was prepared. The protein concentration was measured by the BCA method, followed by electrophoresis, membrane transfer, blocking, and incubation with primary and secondary antibodies [9]. NRF2 XP rabbit mAb, p38 MAPK (D13E1) XP Rabbit mab and phospho-p38 MAPK (Thr180/Tyr182) (D3F9) Rabbit mAb were purchased from CST. Anti-beta actin antibody was obtained from Abcam. Colour development was carried out by using enhanced chemiluminescence (ECL), and grey scale analysis by using Image-Pro Plus 6.0 software (Media Cybernetics Corp, Rockville, MD, USA).

### 2.6. Statistical Analysis

The data of all tests are presented as mean ± standard deviation ($\bar{x}$ ± SD). Data were analysed using SPSS 24.0 software (IBM Corp, Armonk, NY, USA) by one-way ANOVA. As for repeated tests, the results were analysed using repeated-measures ANOVA. The LSD method was used for inter-group comparisons if the variance was equal, and, otherwise, a Tamhane test was used. The criterion for statistically significant was *p* < 0.05.

## 3. Results

### 3.1. Exogenous NTs Affected Body Weight in Ageing SAMP8 Mice

**Figure 1:**
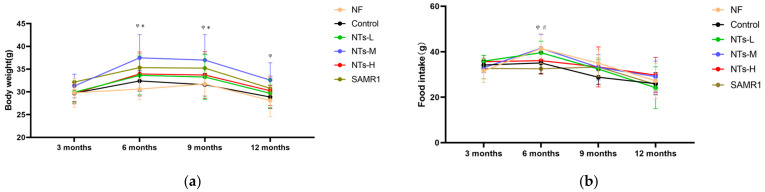
Effect of NTs on the bodyweight and food intake of mice. (**a**) The bodyweight of mice between 3 months and 12 months of age; (**b**) Food intake of mice between 3 months and 12 months of age. ^φ^ Compared to NF, *p* < 0.05; * Compared to Control, *p* < 0.05; ^#^ Compared to SAMR1, *p* < 0.05. The data were analyzed using repeated-measures ANOVA, and the LSD method was used for inter-group comparisons.

During the intervention, 2–3 mice died in each group (Table 1), and the amount of surviving mice at the endpoint of the experiment had no statistical significance in all of the groups (*p* > 0.05). As shown in Figure 1a,b, the bodyweight and food intake in all groups showed no statistical significance at the beginning (*p* > 0.05). Bodyweight increased accompanied with food intake from 3 to 6 months of age, and it gradually decreased from 6 to 12 months of age. Overall, supplementation of mid-dose NTs in the diet of ageing SAMP8 mice significantly increased their bodyweight (*p* < 0.05).

### 3.2. Exogenous NTs Improved the Morphology Structure and Function of the Colon

**Figure 2:**
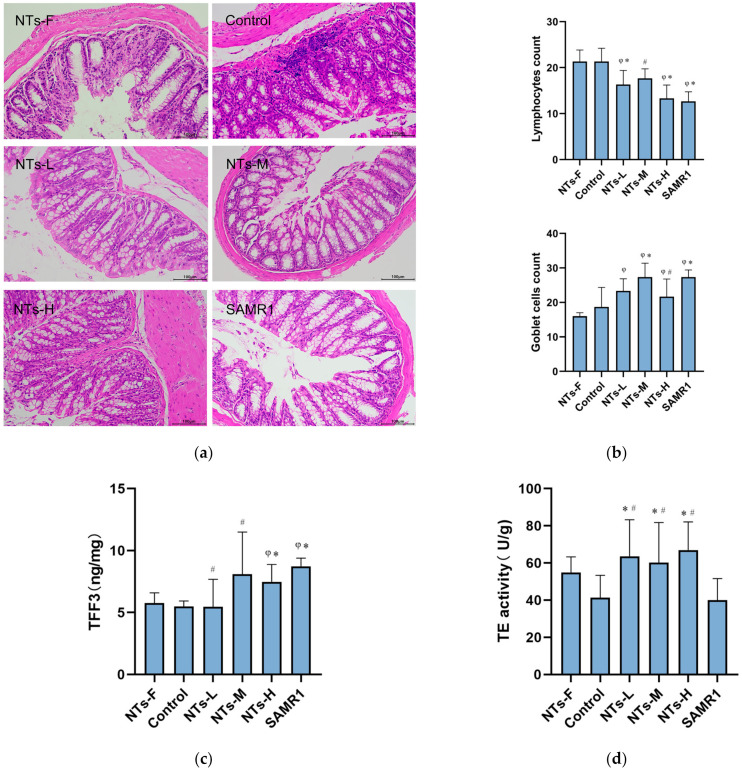
Influence of NTs on the morphological structure in the colon tissue of mice as well as concentration of TFF3 and TE activity. (**a**) H&E staining of colon (magnification: 200×). (**b**) Counting the number of lymphocytes and goblet cells in HE-stained sections, three sections in each group were selected. (**c**) Collect colon tissue of mice at the end of the intervention and store it at −80 °C. 5 mice were randomly selected for the assay. ELISA to detect the concentration of TFF3 in colon tissue. (**d**) Six mice were randomly selected for the assay. ELISA assay for TE activity in colon tissue. ^φ^ Compared to NF, *p* < 0.05; * Compared to control, *p* < 0.05; ^#^ Compared to SAMR1, *p* < 0.05. The result was analyzed using one-way ANOVA, and the LSD method was used for inter-group comparisons.

The result of HE staining showed that the colonic epithelium of the control group was observably aged, showing certain pathological features, such as incomplete mucosa, absence of crypts, disorganized arrangement of intestinal cells, and inflammatory infiltration at the bottom of the crypts (Figure 2a). As shown in Figure 2b, the number of goblet cells declined and the number of lymphocytes increased in the control group (*p* < 0.05). At the same time, the mice in the NTs intervention groups showed some improvement in the morphology and structure of the ageing colon, such as a neater arrangement of the intestinal epithelium, a greater abundance of goblet cells, and the decline of lymphocytes (*p* < 0.05) (Figure 2a,b). The supplementation of exogenous NTs had a beneficial effect on the structure of the ageing colon tissue of mice.

We tested several indicators in the ageing intestine that characterise intestinal function, such as TFF3 and TE, which are the intestinal mucosal protective factors. As shown in Figure 2c, the concentration of TFF3 in the control group was markedly lower when compared with the SAMR1 group (*p* < 0.05), and the concentration of TFF3 in the NTs-H group seemed higher when compared to the control and NTs-F groups (*p* < 0.05). TE in cells is important for cellular DNA repair. Our experiment revealed that compared to the control and SAMR1 groups, TE activity in the NTs intervention groups was notably higher (*p* < 0.05) (Figure 2d). The results suggested that exogenous NTs could increase the concentration of TFF3 and TE in the ageing intestine to improve the intestinal function.

### 3.3. Exogenous NTs Ameliorated Intestinal Immunity and Secretion of Inflammatory Factors in Ageing SAMP8 Mice

**Figure 3:**
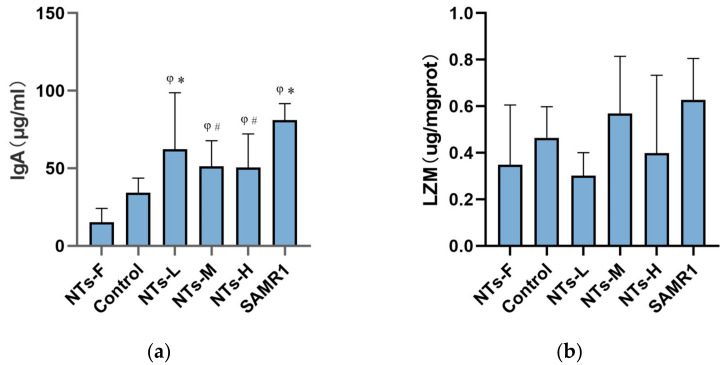
Effect of NTs on the concentration of IgA and LZM in the colon tissue of mice. Six mice were randomly selected for the assays below. (**a**) ELISA to detect intestinal IgA concentration in the colon tissue of mice. (**b**) Self-control method to detect intestinal LZM concentration in mice. ^φ^ Compared to NF, *p* < 0.05; * Compared to control, *p* < 0.05; ^#^ Compared to SAMR1, *p* < 0.05. The data were analyzed using one-way ANOVA, and the LSD method was used for inter-group comparisons.

LZM secreted by pan cells and IgA secreted by plasma cells are vital ingredients of intestinal immune system, and the level of these immune factors can reflect the immune function of the intestine to some extent. As shown in Figure 3a, the concentration of IgA in the control group was signally lower than that in SAMR1 group (*p* < 0.05). Compared to the control group, the concentration of IgA in NTs-L group was markedly higher (*p* < 0.05). In addition, compared with the NTs-F group, the IgA concentration in the colon tissue of the NTs intervention groups and the SAMR1 group were markedly higher (*p* < 0.05). Although we did not find statistical significance in the concentration of LZM in each group (*p* > 0.05), there was a tendency for the LZM concentration in NTs-M group to increase compared to the control group (Figure 3b). Overall, the concentration of IgA in the ageing colon of mice was significantly decreased, while the supplementation of exogenous NTs could obviously increase the concentration of IgA. Hence, exogenous NTs may improve the intestinal immunity.

There is a correlation between immune response and inflammation. We next tested the inflammatory level by measuring the content of inflammatory cytokines of intestine. As shown in Table 2, the content of MCP-1 in NTs-L and NTs-H groups were notably lower compared to control group (*p* < 0.05). Although the concentration of TNF-α and CXCL-1 did not have significance between all groups (*p* > 0.05), the content of TNF-α and CXCL-1 of the NTs intervention groups seemed lower than the control group. We found that exogenous NTs were able to inhibit the secretion of inflammatory factors in the intestine of ageing SAMP8 mice.

### 3.4. Exogenous NTs Might Increase Nrf2 Expression and Activate the p38 Signalling Pathway

**Figure 4:**
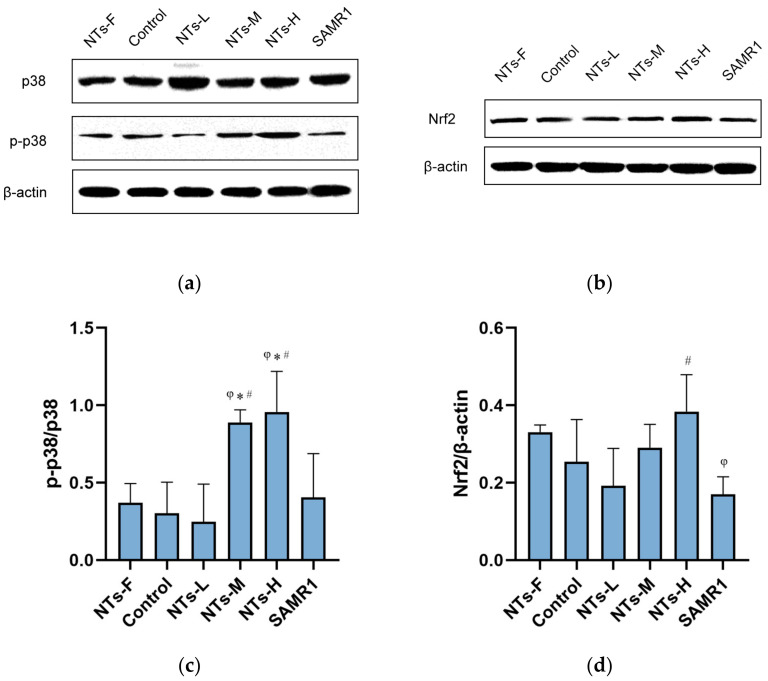
Effect of NTs on the expression of p38, p-p38 and Nrf2 protein in the colon tissue of mice. A total of 3 mice in every group were chosen at random for Western blot assay. (**a**) Immunoblots of p38 and p-p38 proteins. (**b**) Immunoblots of Nrf2 protein. (**c**) The relative amount of activated p38 was expressed as p-p38/p38. (**d**) Nrf2/βactin. ^φ^ Compared with NF, *p* < 0.05; * Compared with control, *p* < 0.05, ^#^ Compared to SAMR1, *p* < 0.05. The data were analyzed using One-way ANOVA, and the LSD test was used for inter-group comparisons.

To explain the mechanism by which exogenous NTs improved the ageing intestine, we examined the expression of Nrf2 and activated p38 proteins. As shown in Figure 4c, the ratio of p-p38/p38 protein in the intestine of mice in NTs-M and NTs-H groups were markedly higher than that in NTs-F group, the control group and the SAMR1 groups (*p* < 0.05). The results indicated that exogenous NTs activated the p38 signaling pathway in the ageing intestine of mice. Although the expression of Nrf2 showed no statistical significance in all groups (*p* > 0.05), it appeared to be higher in the NTs-H group compared with the control group (Figure 4d). This offers the possibility that high-dose NTs increased the expression of Nrf2 protein in the ageing intestine of mice.

## 4. Discussion

This is an important study to explore the influence of long-term exogenous NTs intervention on age-related intestinal inflammation in SAMP8 mice. The structure and function of the intestine changes with age. In this study, the intestine of SAMP8 mice showed obvious ageing features at 12 months of age, and we found that exogenous NTs intervention was able to alleviate the damage caused by ageing. SAMP8 mice lost weight as they aged, but 0.6 g/kg NTs appeared to be able to maintain bodyweight by increasing the food intake of the mice. Exogenous NTs improved the morphology of the senescent colon, and they reduced inflammatory infiltration of the intestine. TFF3 is vital for protecting intestinal mucosa [30], and it has a mucosal repair function [31]. We discovered that the level of TFF3 was notably decreased in the ageing intestine of mice, and 1.2 g/kg NTs improved TFF3 concentration in the intestine. Telomeres are parts of chromosomes, and telomeres can protect chromosomal DNA from damage [32]. Telomerase (TE) is able to lengthen telomeres and reduce DNA replication shortening. It is thought that the amount of TE expressing cells decreases with age, as does the level of TE expression [33]. Meanwhile, treatment targeting TE can slow down ageing [34]. In our study, TE activity was notably higher in low, medium, and high doses of the NTs intervention groups than in the control group. Our study suggested that exogenous NTs were able to increase TE activity in an ageing intestine, so they could promote DNA damage repair in cells.

Ageing can lead to a decline in immunity and the occurrence of inflammation, and it causes systemic diseases in severe cases. As a result, reducing the level of inflammation and improving immunity are important ways to improve health. Some studies have shown that pro-inflammatory factors increase when the senescent intestinal barrier is damaged [35,36]. Similarly, in our experiment, we found a significant decrease in MCP-1 concentration in the intestine of the NTs-L and the NTs-H groups, and this result was consistent with the result of the count of lymphocytes. The above findings showed that the intervention of 0.3 and 1.2 g/kg NTs could clearly limit intestinal inflammation. The inflammatory response is, to some extent, induced by immune cells and their secreted factors, and the inflammatory response becomes a fundamental mechanism of the immune response [37]. One method to target the inflammatory response is to improve the body’s immunity. Mature plasma cells in the intestinal epithelium are capable of producing local antibodies, such as immunoglobulins and anti-microbial peptides (AMPs). Local antibodies are important components of the intestinal immune system. Intestinal immunity declines progressively with age, which is called “immune senescence” [38]. The secretion of immunoglobulin sIgA decreases in the ageing intestine due to the ageing of immune cells [39], and this leads to a decline of intestinal immunity. In this study, we found that the IgA concentration in the ageing intestine of mice clearly decreased, and that it increased in the NTs-L group. The results indicated that 0.3 g/kg NTs could improve intestinal immunity.

Ageing of the intestine is reflected in intestinal inflammation to some extent. Dysregulation of the Nrf2 signaling pathway is now thought to be the pathogenesis of some diseases. Nrf2 activity and its regulatory mechanisms are important for health. The Nrf2 pathway has strong anti-inflammatory activity, and it directly antagonizes the transcription of pro-inflammatory genes to regulate anti-inflammatory functions [40]. Nrf2 activation is significantly lower in older individuals than in younger individuals, and its expression decreases with age [41]. The Nrf2 pathway is also one way to help prevent inflammatory disease as it might maintain intestinal barrier integrity and reduce inflammation through the Nrf2 pathway [42]. The results of this study showed that Nrf2 expression in the intestine of mice in the NTs-H group may increase, so 1.2 g/kg NTs might activate Nrf2 protein expression to regulate the level of intestinal inflammation. Some investigators have found that the p38 MAPK pathway is closely related to physiological pathways, such as cellular oxidative stress, inflammation, and organelle stress [43]. Ageing is associated with decreased activity of p38 MAPK signaling pathway [44], and p38 MAPK signaling may be activated by ribosomal stress in order to protect the gastrointestinal barrier during ageing [45]. The anthocyanins can activate the p38 MAPK pathway in intestinal cells to induce apoptosis, and it subsequently has both antioxidant and anti-inflammatory effects [46]. In addition, the p38 signaling pathway has been found to play an important role in the induction and the suppression of tumors [47]. Some components of the p38 pathway are involved in tumor suppression by controlling cellular responses, such as oncogene-induced senescence, replicative senescence, contact inhibition, and so on. Furthermore, the inactivation of the p38 pathway is more conducive to tumorigenesis. The dual role of the p38 pathway in tumor suppression suggests that we need to focus on specific p38 downstream effectors in order to distinguish the role of the p38 pathway. This study found that the expression ratio of the activated p38 protein in the intestine of mice in the NTs-M and the NTs-H groups increased markedly, which means that 0.6 and 1.2 g/kg NTs could activate the p38 pathway. It has been suggested that a low dose of 0.3 g/kg NTs was unable to activate the p38 pathway, and that doses of 0.6 and 1.2 g/kg NTs had an obvious effect on inflammatory pathways. These results further support the idea that the p38 signaling pathway was activated by exogenous NTs in the intestine, thereby activating other signaling pathways associated with it, such as physiological responses that include apoptosis, autophagy, and cell cycle regulation. In this way, exogenous NTs could regulate intestinal homeostasis.

NTs can be either synthesized in the body or absorbed from outside. The deficiency of dietary NTs affects the growth and the function of the intestine. The rate of intestinal protein synthesis is overtly reduced when NTs are taken out of the diet, and the activity of intestinal enzymes, such as alkaline phosphatase, sucrase, maltase, and lactase, also reduce at the same time. The maturation of intestinal cells is slowed when NTs are deprived, too [18,48]. The ageing intestine requires more NTs from the diet to compensate for the lack of synthesis and absorption. There are very few studies on how the lack of NTs affects intestinal function. However, we found no statistical significance in the intestine of mice in the NTs-F group compared to the control group, although some indicators suggested that the deficiency of dietary NTs might cause adverse effects. Further studies are needed on this issue in the future.

In our experiment, exogenous NTs were added to fodder in order to investigate how exogenous NTs influence the ageing intestine of mice. The findings revealed that exogenous NTs might improve both the structure and the function of an ageing intestine by inhibiting inflammation and promoting immunity. However, due to the limitations of the experimental conditions, the intestinal tissues used in this study were taken after the mice had died, and the intestinal function of the mice was not assessed during the intervention. Furthermore, as the intestinal epithelium is a relatively rapidly renewing tissue, we did not find any significant pathology in the ageing intestine of mice. In future, we will consider extending the duration of the intervention to allow for a more pronounced intestinal senescence, as well as dynamic observation of intestinal function during the experiment. Our future work will also investigate the role of exogenous NTs in other signaling pathways in the ageing intestine.

## 5. Conclusions

In our study, exogenous NTs intervention improved the structure and function of the ageing intestine, such as increasing the amount of goblet cells and the level of intestinal protective factors. Exogenous NTs could inhibit intestinal inflammation by reducing the number of lymphocytes and the level of inflammatory factors, and could increase the level of immune factors to promote intestinal immunity. We also found that exogenous NTs may activate the p38 and the Nrf2 pathways. In conclusion, exogenous NTs have the potential to ameliorate the ageing intestines of mice, and, therefore, its application to nutritional intervention is expected to protect intestinal health in the elderly.

## Figures and Tables

**Table 1:** Description of animals.

Groups	Fodder	Number of Animals	Survival Animal Numbers
Control	Standard food	15	12
SAMR1	Standard food	15	12
NTs-F	Purified food, AIN-93M	15	13
NTs-L	Standard food + 0.3 g/kg NTs	15	14
NTs-M	Standard food + 0.6 g/kg NTs	15	13
NTs-H	Standard food + 1.2 g/kg NTs	15	12

**Table 2:** Effect of NTs on the content of inflammatory cytokines in colon tissue of mice.

Groups	IL-2 (pg/mg)	TNF-α (pg/mL)	MCP-1 (pg/mg)	CXCL-1 (pg/mg)
NTs-F	0.55 ± 0.13	290.74 ± 165.54	41.65 ± 27.30	2.20 ± 2.41
Control	0.43 ± 0.18	279.61 ± 56.87	62.38 ± 27.33	37.85 ± 29.21
NTs-L	0.28 ± 0.12	357.03 ± 241.62	29.72 ± 10.55 *	18.64 ± 12.19
NTs-M	0.38 ± 0.20	188.47 ± 122.16	72.51 ± 41.79	18.45 ± 16.23
NTs-H	0.29 ± 0.33	194.19 ± 110.79	24.85 ± 7.26 *	15.62 ± 4.36 ^φ^
SAMR1	0.48 ± 0.25	460.95 ± 191.85	42.78 ± 24.91	19.25 ± 5.57 ^φ^

NTs supplementation inhibited the secretion of inflammatory factors in the ageing intestine of mice. A total of 5 mice were randomly selected for the IL-2 assay, and 6 mice were randomly selected for the TNF-α, MCP-1, and CXCL-1 assays. ^φ^ Compared with NF, *p* < 0.05; * Compared with control, *p* < 0.05. The data were analyzed using one-way ANOVA, and the LSD or Tamhane test was used for inter-group comparisons.

## Data Availability

The data presented in this study are available on request from the corresponding author. The data are not publicly available due to privacy.
